# Doing the right thing! A model for building a successful hospital-based ethics committee in Nunavut

**DOI:** 10.3402/ijch.v72i0.21326

**Published:** 2013-08-05

**Authors:** Madeleine Cole, Gwen Healey

**Affiliations:** 1 Qikiqtani General Hospital, Iqaluit, Nunavut, Canada; 2 Qaujigiartiit Health Research Centre, Iqaluit, Nunavut, Canada

**Keywords:** health, ethics, Inuit, Arctic, Nunavut, research

## Abstract

**Background:**

There exists a need throughout the North to increase capacity to address issues of health ethics and for community members to better understand and share their perspectives on this topic. Ethics comes down to weighing rights and wrongs, evaluating differing needs and understandings, acknowledging the many shades of grey and doing our best to come up with the just, fair and moral approach to the question at hand. Northern regions must collaborate to share capacity, successes and experiences in order to meet the unique needs of northern health care institutions and move forward on this issue. While guidelines for ethical research with indigenous populations exist, little has been published about an Inuit approach to health ethics more broadly.

**Design:**

To fill a critical need and to meet accreditation standards, the Qikiqtani General Hospital (QGH) in Iqaluit, Nunavut, Canada, is in the process of building an Ethics Committee. Capitalizing on partnerships with other bodies both in northern and southern Canada has proved an efficient and effective way to develop local solutions to challenges that have been experienced both at QGH and other jurisdictions.

**Methods:**

The Ottawa Hospital Ethics Office and the active ethics committee at Stanton General Hospital in Yellowknife, NT, contributed expertise and experience, and helped provide some direction for the QGH ethics committee. At the local level, based on our shared commitment to health care ethics, the Qaujigiartiit Health Research Centre is an invaluable partner whose parallel efforts to develop a northern Health Research Ethics Board (REB) gives great synergy to the QGH Ethics Committee.

**Results:**

Passion and commitment, as well as administrative support and endorsement from health care leaders, are the aspects of successful initiatives that we have identified to date. Using the information from both the experiences of other partners, as well as information gathered at a retreat held in Iqaluit in September 2011, we are working to develop a model for the QGH ethics committee that incorporates multi-level perspectives, from that of community to that of front-line worker.

**Conclusion:**

Ideally, the scope of the QGH Ethics Committee will grow over time to include ethics education, facilitation of clinical ethical consults, ethical review of policy, advice on governance issues and involvement and support of an external northern Health REB.

Nunavut, meaning “our land” in Inuktitut, is Canada's newest and largest territory and has a mere 30,000 people spread across 1.9 million square kilometres. Delivering health care is an on-going challenge for many reasons, as is building the capacity to do it both well and ethically. There is a rapid turnover of mostly non-Inuit health care professionals who work in the many small health centres and in the territory's only hospital. In contrast, Inuit, the first peoples of the Canadian Arctic, make up about 85% of the population of Nunavut. Inuit culture and our vast geography may be the 2 main factors creating unique opportunities and challenges when considering the ethical delivery of health care in Nunavut.

Qikiqtani General Hospital (QGH) is based in the capital of Iqaluit, and patients requiring tertiary care or specialized testing are sent to southern Canada to centres such as Ottawa, Winnipeg and Edmonton. The great distances that people must travel to get any form of specialized health care or diagnostic care leads to a number of ethical dilemmas specific to Nunavut (and similar remote settings). One-third of Nunavut's health care budget is spent on moving people to a site that can provide the care they need. A number of ethical issues are inherent in the distribution of finite resources for medical care. For example, a lack of breast and colon cancer screening programs and the medical travel escort policy. Ethical dilemmas are also presented by Nunavut's policies related to birthing. Policies require health practitioners to balance patient risk with autonomy, and most women are required to leave their communities a month before their due date to await labour away from their families in a medical boarding home in Iqaluit (or out of the territory) where more advanced medical care is available than in a community health centre. While this practice has reduced the number of perinatal maternal and infant deaths and enforced a standard of care more closely in line with that of the rest of Canada, the practice separates pregnant mothers from their spouses and families, which has been identified as a problem by community members.

## Hospital-based ethics

For over 20 years, having some form of mechanism to address ethical conflicts that arise in the care of patients has been a requirement of hospital accreditation in North America (1). Hospital-based health care ethics committees and programs most often provide ethics education, consultation services, and advise on organizational ethics (policy development and governance). Some ethics committees also act as de facto research ethics boards, or work closely with their regional Research Ethics Boards (REB).

Within the health care system, opportunities for ethical reflection abound. Beginning and end-of-life care, capacity concerns, advance directives and other aspects of primary health care delivery each present opportunities for ethical reflection and discussion. Ethical dilemmas also arise in other areas such as health economics, resource distribution, public health (e.g. mandatory TB treatment, STI contact tracing), when and how to involve medical and allied health care professional learners in clinical care, policy development and relationships with industry.

Qaujigiartiit Health Research Centre is a community-driven, northern led, health and wellness research centre that facilitates action on community-identified health research priorities in Nunavut. The centre works to enable health research to be conducted locally, by northerners, and with communities in a supportive, safe, culturally-sensitive and ethical environment. Qaujigiartiit works within a model that is respectful and mindful of Inuit and western science epistemologies and methodologies to implement research projects for the pursuit of good health for all Nunavummiut (the people of Nunavut). On-going collaboration with Qaujigiartiit has been one important way to maintain relationships with other community-based health organizations outside of the hospital with similar goals and motivations.

## Methods: our story

To foster a workplace that encourages ethical reflection, and to meet accreditation standards, in 2011 a small contingent of committed health care workers began the process of building an ethics committee at QGH. Rather than outsourcing ethics to “experts” thousands of kilometres away, it was strongly felt that we needed to build our local capacity. As described by Swenson and Miller (2), “By having an ethics committee, a hospital sends a very different message: moral discourse is a community enterprise, an exercise in social intelligence that must not be divorced from everyday life and practice.”

One of the core areas of focus for the QGH Ethics Committee was to build the committee through partnership and collaboration. Looking to external expertise both locally and farther afield was critical to success. The planning expertise and research ethics focus of an Iqaluit-based organization, Qaujigiartiit Health Research Centre (and its executive director co-author, Gwen Healey) helped guide some of the work on actualizing the committee and building our terms of reference. Dr. Tom Foreman and Mike Kekewich of the Office of Organizational and Clinical Ethics at The Ottawa Hospital remain an excellent source of advice and mentorship in the process of developing a functioning ethics committee in the far north. Members of the Ethics Committee at Stanton General Hospital in Yellowknife, Canada, also graciously shared their model, terms of reference and insights into how to build a thriving committee.

## Results

One early challenge faced in the creation of the ethics committee was to establish the scope and membership of the committee. Over the course of a 2-day ethics retreat, we attempted a form of engagement with like-minded community members and organizations, which focused on building the committee (day 1) and on building more capacity for research ethics review (day 2). Representatives from Inuit government and non-government organizations were invited, as were representatives from Nunavut Arctic College, and health care representatives from the mental health sector and the public health department. With funding from the Canadian Institutes of Health Research, our Ottawa Hospital bioethicist colleagues were able to visit Iqaluit and the QGH to educate, learn about and lead discussions on the role of health care ethics in our institutions. It was eventually decided that membership of the Hospital Ethics Committee would include interested workers at the QGH, that all hospital staff would be invited to attend meetings, and that persons external to the hospital might be invited (or themselves ask) to attend meetings as required. Further, the committee would at this stage remain accountable to the community of patients served by the QGH specifically, but would act as a resource when called upon by other health centres in Nunavut and to the Department of Health and Social Services more broadly.

**Fig. 1:**
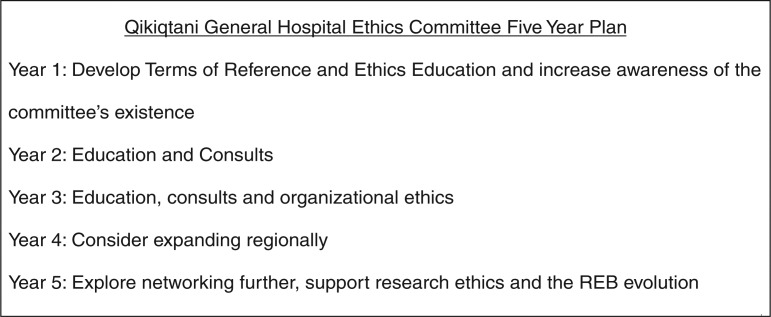
Qikiqtani general hospital ethics committee 5-year plan.

In the 2-day ethics retreat, a draft 5-year plan was developed for the QGH Ethics Committee to allow for an incremental increase in capacity (Fig. 1).

Kaczynski stated, “The goal of ethics committees should be changing the very culture of a healthcare institution rather than changing behavior in an individual case” (3). While the QGH Ethics Committee does intend to grow its internal capacity to provide competent ethics case consultation, our intentions are farther reaching.

Subsequent to the planning stages for the ethics committee (partnership building and 2-day ethics retreat), in the first year of regular meetings, the QGH Ethics Committee accomplished a number of things. These include: developing a Terms of Reference; sending 2 nurses to an external ethics training workshop; holding an Ethics case rounds dinner; founding a Health and Ethics Book group; and supporting Ethics educational rounds on a few occasions with presentation and discussion (advanced care directives, capacity assessment, physician assisted death, sex selective abortion, consent and more). Despite organizational ethics being a third year goal for the committee, spurred by real-life hospital-based ethical dilemmas, a new and relevant policy on treatment of minors at the hospital was also developed.

## Discussion: cultural safety

Moving forward, we believe it will be important to make time for concrete reflection on how and if the cultural, socio-political and historical landscape of Nunavut, as well as Inuit specific social determinants of health, affect aspects of our ethical work as health service providers and as a formalized health ethics committee. The perspectives of Inuit towards health ethics should not be assumed and as has been noted, little has been published to date.

Wayne Clark, a Nunavut land claim beneficiary, has touched on many ethical issues in his thoughtful thesis on Inuit cultural considerations when building electronic health records and communicating ownership of the information therein (4). He offers the now disbanded National Aboriginal Health Organization's definition of cultural safety as “what is felt or experienced by a patient when a provider communicates in a respectful and comprehensive way that empowers the patient in decision-making and builds a relationship where the patient and provider work together to optimize a maximum level of care”. Articulated first as a nursing education concept in New Zealand to improve care for the Maori (5, 6), cultural safety is a very useful lens for working with diverse populations and especially when working with Indigenous populations who have experienced colonization. In Clark's writing on positionality in research (and how the researcher can be thought to be part of the research rather than separate from it), parallels can be drawn in the QGH ethics committee. As mostly non-Inuit members of a hospital ethics committee that serves a majority Inuit population, we need to ask ourselves: “Are we contributing to Inuit cultural safety?”

**Fig. 2:**
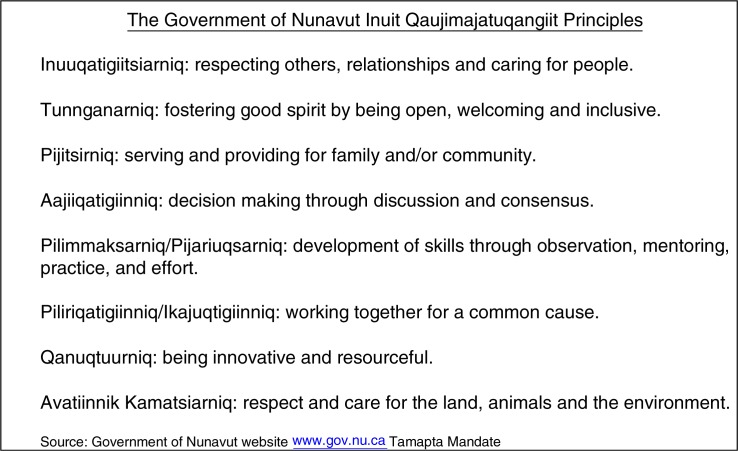
The Inuit Qaujimajatuqangiit principles that provide the foundation for the Government of Nunavut (7). Source: Government of Nunavut website www.gov.nu.ca Tamapta Mandate.

The Government of Nunavut, which was formed in 1999 as the result of the Nunavut Inuit Land Claims agreement has strong goals when it comes to Inuktitut language preservation and has also articulated 8 principles of Inuit Qaujimajatuqangiit (IQ) that relate to governance and the goals of the Nunavut Government (Fig. 2).

Inuit concepts that articulate values and ways of being, these 8 principles of IQ are intended to guide the work of all government departments including the Department of Health and Social Services. IQ principles emphasize family, community and collaboration: the Inuit worldview is *we* more than *me*. There is less fixation on personal autonomy which is central to most “western” approaches to health ethics; quite possibly the presumed good and ethical approaches to end-of-life care in the majority of western society (and disclosure of information and much more) might be understood differently from an Inuit perspective. There are incredible opportunities for learning from Inuit hospital co-workers, elders and friends. Acculturation affects us all in different ways. Within our institutions, such as hospitals and health centres, ethically sound health care requires concrete acknowledgement and respect for (as well as curiosity and celebration of) the traditional and contemporary ways of the Inuit.

The Government of Nunavut Department of Health and Social Services has an IQ specialist on staff and there is an open invitation to this knowledgeable Inuk individual to attend all QGH Ethics Committee meetings. The Terms of Reference state that the committee “will also work to foster community partnerships in order to keep a multicultural perspective that respects and incorporates the principals of Inuit Qaujimajatuqangiit.” While there is no structural or procedural guidance at this stage to clarify how this might be achieved, we have built the committee through partnership and will continue to collaborate with others.

## Next steps

Ethics, broken down to its basic principles, is about weighing rights and wrongs, evaluating differing needs and understandings, acknowledging when medical decisions are difficult or enter into a “grey” area, and doing our best to navigate ourselves toward the just, fair and moral approach to the question at hand. Through ethics education, and with humility, we will strive to support hospital staff, and the patients and community we serve, to improve all of our understandings of the various principles of health ethics, and of the rights and responsibilities of people seeking and delivering health care.

When reflecting on the evolution of the QGH Ethics Committee and moving toward evaluating its successes, the oft quoted expression of an early English jurist comes to mind: “Not only must Justice be done; it must also be seen to be done” (8). It is our belief that through dialogue and knowledge sharing there is room to engage Nunavummiut in learning opportunities and discussions on such basic topics as informed consent and confidentiality as well as more nuanced ethical principles, such as end-of-life decision-making. Education has been highlighted as one of the first priorities for the QGH Ethics Committee and this work, and if implemented as envisioned, will involve multi-directional knowledge-sharing and involve multiple community, health care and policy perspectives.

## Conclusion

In summary, over the past 2 years, committed staff moved purposefully with the support of senior management to create a Health Ethics Committee at the QGH. Our greatest and most interesting challenge may be the meaningful exploration and inclusion of contemporary Inuit ways of knowing and understanding wellness as they relate to health ethics. Ethics education, case consults and organizational ethics will be the main foci of the committee. We will also collaborate with Qaujigiartiit Health Research Centre to support the development of a northern REB. It is our goal to be inclusive, interesting and relevant. The QGH Ethics Committee will continue to foster an ethical workplace, and contribute to improving the health and well-being of the people of Nunavut.
